# MRI-derived radiomics to guide post-operative management of glioblastoma: Implication for personalized radiation treatment volume delineation

**DOI:** 10.3389/fmed.2023.1059712

**Published:** 2023-01-19

**Authors:** S. Chiesa, R. Russo, F. Beghella Bartoli, I. Palumbo, G. Sabatino, M. C. Cannatà, R. Gigli, S. Longo, H. E. Tran, L. Boldrini, N. Dinapoli, C. Votta, D. Cusumano, F. Pignotti, M. Lupattelli, F. Camilli, G. M. Della Pepa, G. Q. D’Alessandris, A. Olivi, M. Balducci, C. Colosimo, M. A. Gambacorta, V. Valentini, C. Aristei, S. Gaudino

**Affiliations:** ^1^Department of Radiology, Radiation Oncology and Hematology, Fondazione Policlinico Universitario “A. Gemelli” IRCCS, Rome, Italy; ^2^Department of Diagnostic Imaging, Oncological Radiotherapy and Hematology, Institute of Radiology, Fondazione Policlinico Universitario “A. Gemelli” IRCCS, Rome, Italy; ^3^Radiation Oncology Section, University of Perugia, Perugia, Italy; ^4^Perugia General Hospital, Perugia, Italy; ^5^Department of Neurosurgery, Mater Olbia Hospital, Olbia, Italy; ^6^Department of Neurosurgery, Agostino Gemelli University Polyclinic (IRCCS), Rome, Italy; ^7^Medical Physics, Mater Olbia Hospital, Olbia, Italy

**Keywords:** radiomic, glioblastoma, target volume definition, heterogeneity, precision medicine

## Abstract

**Background:**

The glioblastoma’s bad prognosis is primarily due to intra-tumor heterogeneity, demonstrated from several studies that collected molecular biology, cytogenetic data and more recently radiomic features for a better prognostic stratification. The GLIFA project (GLIoblastoma Feature Analysis) is a multicentric project planned to investigate the role of radiomic analysis in GB management, to verify if radiomic features in the tissue around the resection cavity may guide the radiation target volume delineation.

**Materials and methods:**

We retrospectively analyze from three centers radiomic features extracted from 90 patients with total or near total resection, who completed the standard adjuvant treatment and for whom we had post-operative images available for features extraction. The Manual segmentation was performed on post gadolinium T1w MRI sequence by 2 radiation oncologists and reviewed by a neuroradiologist, both with at least 10 years of experience. The Regions of interest (ROI) considered for the analysis were: the surgical cavity ± post-surgical residual mass (CTV_cavity); the CTV a margin of 1.5 cm added to CTV_cavity and the volume resulting from subtracting the CTV_cavity from the CTV was defined as CTV_Ring. Radiomic analysis and modeling were conducted in RStudio. Z-score normalization was applied to each radiomic feature. A radiomic model was generated using features extracted from the Ring to perform a binary classification and predict the PFS at 6 months. A 3-fold cross-validation repeated five times was implemented for internal validation of the model.

**Results:**

Two-hundred and seventy ROIs were contoured. The proposed radiomic model was given by the best fitting logistic regression model, and included the following 3 features: F_cm_merged.contrast, F_cm_merged.info.corr.2, F_rlm_merged.rlnu. A good agreement between model predicted probabilities and observed outcome probabilities was obtained (*p*-value of 0.49 by Hosmer and Lemeshow statistical test). The ROC curve of the model reported an AUC of 0.78 (95% CI: 0.68–0.88).

**Conclusion:**

This is the first hypothesis-generating study which applies a radiomic analysis focusing on healthy tissue ring around the surgical cavity on post-operative MRI. This study provides a preliminary model for a decision support tool for a customization of the radiation target volume in GB patients in order to achieve a margin reduction strategy.

## 1. Introduction

Glioblastoma (GB) continues to be the most common and threatening primary brain tumors in adults and despite a multimodal treatment (maximum safe surgical resection followed by adjuvant radio-chemotherapy with Temozolomide) the prognosis remains poor, with a median overall survival (OS) of 14.6 months and a median progression free survival (PFS) of 6.9 months (1). In spite of decades of research, our knowledge of this neoplasm is still limited. This bad prognosis is primarily due to intra-tumor heterogeneity, demonstrated also from several studies that collected molecular biology and cytogenetic data for a better prognostic stratification of glioblastoma.

The implementation of these markers, however, depends in routine clinical practice on surgical tissue (2). On the contrary, the use of medical imaging, as a non-invasive tool to derive prognostic factors that can predict outcome such as survival, PFS, and response to therapy, is becoming increasingly popular. The images can be described not only qualitatively in order to highlight the presence of necrotic, edemigenous, malignant, suspected or metabolically active areas, but also quantitatively in order to generate numbers that become real measurable data (3–5).

Radiomics (6) is the process that involves the high-throughput extraction of quantitative features by computing local macro and micro-scale morphologic changes in texture patterns (e.g., roughness, image homogeneity, regularity, edges) with the intent of creating mineable databases from radiographic images.

Some experiences with glioblastoma are reported *via* radiomics approaches to predict tumor’s histological features (7), progression (8), grade, treatment response (9), or even overall survival (10–13).

Magnetic resonance imaging (MRI) is the imaging modality for characterizing GB in these studies and generally has an integral role in diagnosis, response assessment, surveillance and radiation treatment, especially for defining the volume of irradiation (14).

Defining the optimal target volume for GB is still a challenge and represents a balance between minimizing treatment related toxicity, while ensuring efficacy in terms of tumor control and allowing a re-irradiation approach. The recent ESTRO-ACROP guidelines in macroscopically resected GB recommend to add an isotropic margin of 2 cm, adjusted to anatomical border, to resection cavity plus any residual enhancing tumor on contrast-enhanced T1 weighted MRI, without considering the peri-tumoral oedema.

This size of safety margin had traditionally been defined around 2–3 cm based on early anatomic and clinical research. In fact, the recurrences reported in several studies are mainly central, in field or marginal (80–90%) with 10–20% of lesions outsides the irradiated field (15, 16).

Several studies have been conducted to identify look for strategies of margin reduction, such as peritumoral zone investigation, the analysis of pattern of recurrence (15) or integration between different imaging methods (17), but no clear indication of reducing margin is yet available (18–22).

In light of all these considerations, the GLIFA project (GLIoblastoma Feature Analysis) is a multicentric project planned to investigate the role of radiomic features in GB management. In particular, in this study we aim to verify whether there are any radiomic features in the tissue around the resection cavity which may guide the target volume delineation allowing a margin reduction strategy toward a personalized medicine approach (23).

### 1.1. Patients selection

This is a multicentric retrospective study approved by the ethics committees of Institutions involved. All procedures performed were in accordance with the ethical standards of the institutional and/or national research committee and with the 1964 Helsinki declaration and its later amendments or comparable ethical standards.

All adult patients, with histologically proven glioblastoma Isocitrate dehydrogenase (IDH) wild-type underwent total or near-total resection of the enhancing tumor, followed by standard radio-chemotherapy and adjuvant chemotherapy (1), who have performed MRIs according to a timeline protocol of image acquisition shared among the project participants, were considered eligible in this study (Table 1). All MRIs must contain at least the post-contrast T1-weighted sequences and T2-weighted Fluid Attenuated Inversion Recovery (FLAIR) and relative images must be available in the required imaging protocol descriptions from Digital Imaging and Communication in Medicine, or DICOM format (24).

**TABLE 1:** Eligibility criteria for GLI.F.A. Project.

Inclusion criteria	Exclusion criteria
– Histological diagnosis of GB > 18 yrs; – ECOG performance status <4; – Total or near-total resection; – Platelet counting > 100 × 109/L; – Hb > 11 g/L; – GB > 4000/mm^3^; – Neutrophils > 1900/mm^3^; – Total bilirubin and alkaline phosphatase at less than 1.25 normal concentration; – Informed consent that documents that the patient has been informed in a way that is clear and comprehensible to him and that fits all aspects of the study.	– Biopsy – Degenerative neurological diseases or other neuropsychiatric disorders; – Pregnancy status; – Respiratory failure; – Immunodepression status; – Chronic renal failure.

ECOG, Eastern Cooperative Oncology Group.

Patients, clinical data and MRI data of GB were obtained from three centers (Università degli studi di Perugia e Azienda ospedaliera di Perugia; Fondazione Policlinico Universitario Policlinico Agostino Gemelli, IRCSS; Mater Olbia Hospital).

Data were collected from patients treated from 2016 to October 2020, with total or near total resection, who completed the standard adjuvant treatment, with at least 9 months of follow-up and for whom we had post-operative images available for features extraction.

The MRIs of these patients were examined and patients whose diagnostic images were blurred or with some of the required sequences missing were excluded from the contouring phase.

### 1.2. Image acquisition and segmentation

Imaging was performed on 1.5 T MRI unit from different manufactures (Philips Medical Systems, SIEMENS, GE Medical Systems).

One sequence was included in the current study: gadolinium (Gd) enhanced T1-weighted FSPGR (T1c). The images were acquired with the following imaging parameters: slice thickness 4–5 mm, pixel spacing 0.35–0.90 mm.

The images were loaded in a radiation therapy delineation console (Eclipse, Varian Medical Systems, Palo Alto, CA, USA) and in the open-source software 3D Slicer for the definition of regions of interest (ROI).

Manual segmentation was performed on post gadolinium T1w MRI sequence by cooperation of 2 radiation oncologists expert in the management of brain cancer, with at least 10 years of experience (SC, FB), and all cases were individually reviewed by a neuroradiologist with at least 10 years of experience (SG, RR).

**FIGURE 1:**
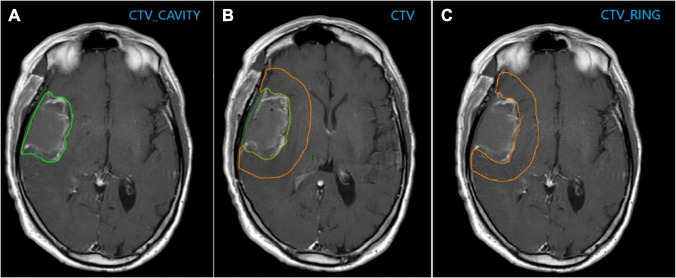
**(A)** CTV_cavity: Surgical cavity ± post-surgical residual mass; **(B)** CTV: CTV_cavity + 1.5 cm; **(C)** CTV_Ring: CTV–CTV_Cavity.

The ROI considered for the analysis were the following: the surgical cavity ± post-surgical residual mass clinical target volume_cavity (CTV_cavity); a margin of 1.5 cm was added to CTV_cavity to obtain the CTV and the volume resulting from subtracting the CTV_cavity from the CTV was defined as CTV_Ring (Figure 1).

### 1.3. Radiomic feature extraction

Radiomic features were extracted from the CTV_Ring using MODDICOM, an open-source R library developed for radiomic feature extraction (25). This software was validated and calibrated within the Image Biomarker Standardization Initiative, which aimed to standardize the definition and computation of radiomic features among different software implementations (26).

In total, 226 radiomic features belonging to different feature families were extracted for each CTV_Ring. 17 statistical features provided statistical measures of the gray-level histogram of the ROI; 14 morphological features provided morphological descriptors of the ROI; 195 textural features described properties of the local distribution of the gray levels within the ROI based on co-occurrence of gray levels, consecutive sequence of pixels or zones with the same gray level (27).

### 1.4. Radiomic feature selection and radiomics modeling

Radiomics analysis and modeling were conducted in RStudio (R version 3.6.3). Z-score normalization was applied to each radiomic feature before further analysis.

We generated a radiomic model using the features extracted from the CTV_Ring to perform a binary classification and predict the PFS at 6 months. Class 1 represented PFS below or equal to 6 months, while class 0 represented PFS above 6 months.

Feature selection was implemented to reduce the number of variables included in the model and prevent overfitting. A univariate analysis was performed using the Wilcoxon-Mann-Whitney statistical test, which tested the statistically significant difference between the two classes for each radiomic feature. A significance level of 0.05 was set for the univariate analysis. The collinearity of the statistically significant features was assessed by computing the Pearson cross-correlation coefficient. We set a threshold of 0.9 for the Pearson coefficient to exclude collinear (highly correlated) features.

Different logistic regression models were generated using the selected features. The best fitting model was determined with a stepwise feature selection according to the Akaike Information Criteria (28), to compromise between model fitting goodness and model complexity.

### 1.5. Radiomic model performance and validation

The internal calibration of the proposed model was evaluated by producing the calibration plot, reporting model predicted probabilities against observed outcome probabilities, and by means of the Hosmer and Lemeshow goodness-of-fit statistic. A *p*-value > 0.05 indicated that there was no statistically significant difference between model predicted probabilities and observed outcome probabilities (29).

The discrimination performance of the proposed model was assessed by calculating the area under the curve (AUC) of the receiver operating characteristic (ROC) curve, and by computing the classification evaluation metrics.

The 95% confidence interval (CI) for the AUC was yielded by performing 2000 stratified bootstrap resampling. Sensitivity, specificity, positive and negative predictive values (PPV, NPV) were computed after defining the probability threshold as the best cut-off according to the Youden’s index method. The 95% CI of these evaluation classification metrics was obtained by adopting the Jeffreys method for small sample sizes (30).

A 3-fold cross-validation repeated five times was implemented for internal validation of the model. Mean and standard deviations of the evaluation classification metrics were calculated over the five repetitions (31, 32).

## 2. Results

### 2.1. Patient population

**FIGURE 2:**
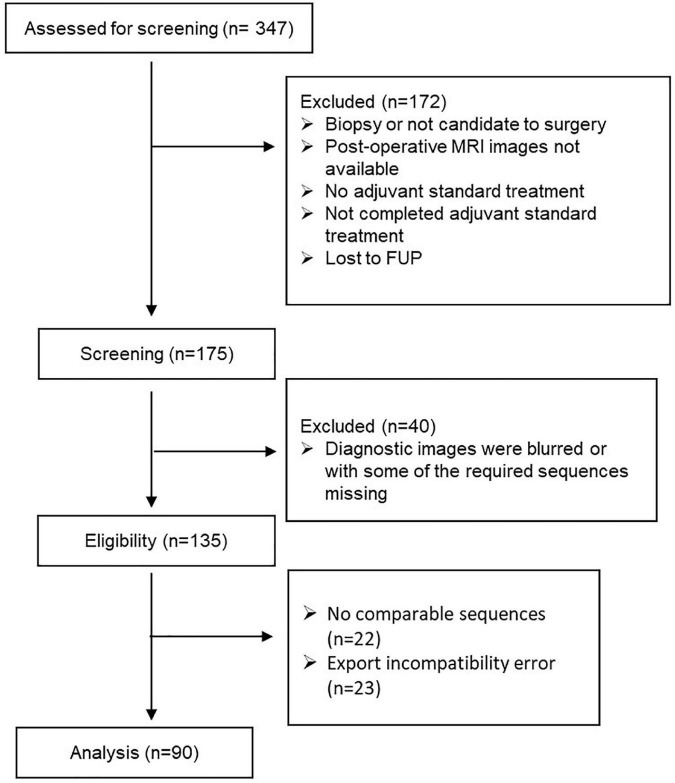
Patients’ selection.

From January 2016 to October 2020, we collected consecutive 347 newly pathologically confirmed patients with GB and screened these cases (Figure 2).

90 patients were considered to retrospectively analyze the pattern of radiomic features.

Patients’ characteristics are reported in Table 2.

**TABLE 2:** Clinical data characteristics of patients with glioblastoma (GB) (*n* = 90).

Characteristics	*n* (%)
**Gender**	
Male	62 (68,9%)
Female	28 (31,1%)
**Age**	
Median	61,7 yrs
Min	80 yrs
Max	39 yrs
<50 yrs	12 (13, 3%)
≥50 yrs	78 (86, 7%)
**MGMT-gene metylathion**	
Not	37 (41, 1%)
Yes	48 (53,3%)
NA	5 (5, 6%)
**Type of surgery**	
GTR	23 (25, 6%)
STR	67 (74, 4%)
**IDH**	
IDH wild-type	100 (100%)
**PFS**	
PFS ≤ 6 months	30 (33, 3%)
PFS > 6 months	60 (66, 7%)

GTR, gross total resection; STR, subtotal resection; IDH, Isocitrate dehydrogenase; MGMT, methylguanine-DNA methyl-transferase; yrs, years.

### 2.2. Development and validation of radiomic model

Based on the Wilcoxon–Mann–Whitney statistical test, 48 out of the extracted 226 radiomic features showed a statistically significant difference between the two classes. Following the correlation analysis with the Pearson coefficient, 12 out of the 48 remaining features were retained for the model development phase. The proposed radiomic model was given by the best fitting logistic regression model, and included the following 3 features: F_cm_merged.contrast, F_cm_merged.info.corr.2, F_rlm_merged.rlnu.

**FIGURE 3:**
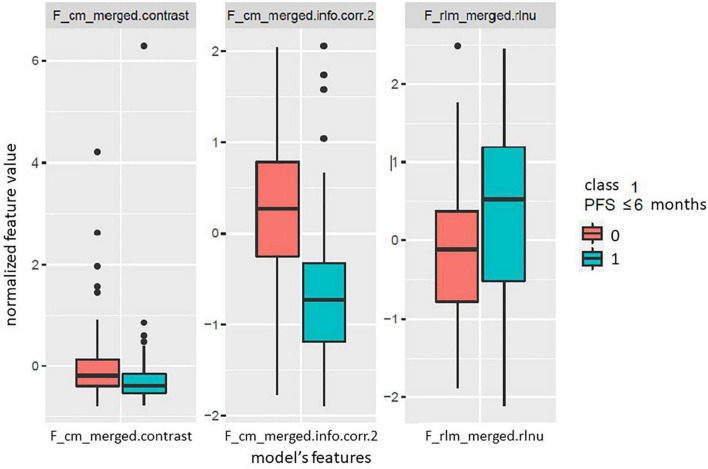
Boxplots of the radiomic features included in the developed logistic regression model for the two classes of the outcome. Class 1 (cyan) indicates progression free survival (PFS) below or equal to 6 months, while class 0 (red) indicates PFS above 6 months.

The boxplots represented in Figure 3 show the distribution of the selected radiomic features used in the model for the two classes of outcome. Table 3 reports the estimated model coefficients and the statistically significant *p*-values (*p*-value < 0.05). The feature F_cm_merged.info.corr.2 which presented no overlap of the interquartile ranges of the two classes, as shown in Figure 3, was also associated to the most statistically significant *p*-value of the model coefficients.

**TABLE 3:** Model coefficients and statistically significant *p*-values.

	Estimated model coefficient	Standard error	*P*-value
Intercept	−0.92	0.27	<0.001
F_cm_merged.contrast	0.89	0.36	0.013
F_cm_merged.info.corr.2	−1.10	0.34	0.0012
F_rlm_merged.rlnu	0.81	0.33	0.014

**FIGURE 4:**
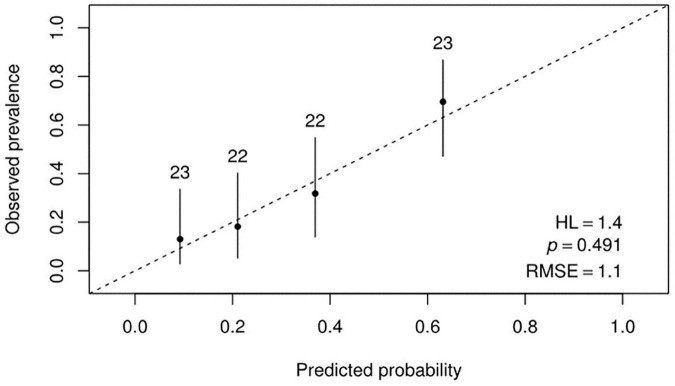
Calibration plot reporting the observed probabilities against the model predicted probabilities.

**FIGURE 5:**
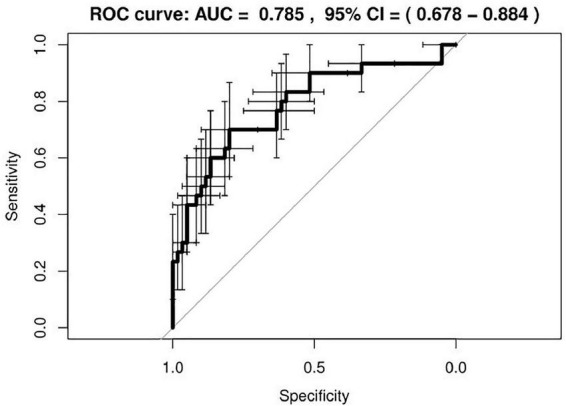
Receiver operating characteristic (ROC) curve of the developed radiomic model. The bars indicate the 95% confidence intervals (CI) for sensitivity and specificity.

A good agreement between model predicted probabilities and observed outcome probabilities was obtained, as showed in the calibration plot (Figure 4) and as indicated by the *p*-value of 0.49 resulting from the Hosmer and Lemeshow statistical test. Figure 5 represents the ROC curve of the model with an AUC of 0.78 (95% CI: 0.68–0.88). The discrimination performances of the model for the binary classification are reported in Table 4 for model fitting and internal validation. The cross-validation confirmed the performances obtained during model fitting with a slight or no decrease of the metrics, suggesting that no overfitting had occurred. Specifically, the specificity decreased from 0.80 during model fitting to 0.75 for the cross-validation, while the NPV remained stable at 0.84.

**TABLE 4:** Model discrimination metrics for model fitting and internal validation with cross-validation.

	AUC	Sensitivity	Specificity	PPV	NPV
Model fitting	0.78 (0.68–0.88)	0.70 (0.52–0.84)	0.80 (0.68–0.89)	0.64 (0.47–0.78)	0.84 (0.73–0.92)
Cross-validation	0.79 (0.04)	0.70 (0.08)	0.75 (0.12)	0.60 (0.15)	0.84 (0.03)

Model fitting presents the 95% confidence interval (CI) of the metrics in brackets. Cross-validation presents mean and standard deviation values (in brackets). PPV, positive predictive value; NPV, negative predictive value.

## 3. Discussion

The emerging big challenge in the field of medical research is to identify multimodal predictive/prognostic factors (clinical, imaging and molecular data) and integrate them in a quantitative manner to provide prediction models that estimate patient outcomes as a function of the possible decisions toward an individualized or personalized medicine.

In the last years, the main effort of radiology research has been focused on quantifying imaging variations trying to understand their clinical and biological implications.

Radiomics uses high-throughput radiomic features and mathematical models to quantify tumor characteristics, allowing the non-invasive capture of microscale information hidden within medical imaging features undetectable by the human eye and add value to clinical visual perception by exposing underlying pathophysiology, including intra-tumoral heterogeneity (29, 33–35).

To date the application of radiomics in GM setting has shown considerable progress in demonstrating that it can be a tool capable of deriving much information, with implications in diagnostics, such as differentiating tumors based on texture analysis, differentiating treatment effects (radiation necrosis, pseudo-progression) and tumor recurrence, in prognosis such as survival stratification (1–4, 34–38) and applications in the choice of optimal therapy (39–41), e.g., stratification of response to anti-angiogenic treatment for recurrent glioblastoma.

Most radiomics studies have focused on analyzing features extrapolated from pre-operative MRI by studying the macroscopic site of the tumor, using ROIs such as tumor enhancement (ET), non-enhancement, tumor/necrosis (NET), and edema (ED).

Few studies (42, 43) have suggested that heterogeneity extends beyond the tumor margins into the peritumoral brain region (PBR), suggesting that the interaction of specific cells (i.e., glioma cells, vascular endothelial, neuroglial and microglial cells) (44, 45) and molecular events in the PBR contribute to tumor infiltration, blood-brain barrier impairment and micro-vascularization and ultimately affect overall survival in GB.

There has been also an increasing interest in understanding the role of the PBR in molecular pathogenesis, as the residual cells along the resection margin and in the surrounding region can represent resistant and rapidly proliferating clones (43), which can lead to disease recurrence (46).

On the other hand, as we know, the anatomy of the brain can be significantly altered after surgery and the characteristics of the tissue surrounding the surgical cavity can be affected by postoperative changes such as gliosis, ischemia, blood products and can be the site of resistant and rapidly proliferating clones. After all, in radiotherapy, postoperative MRI is the imaging of choice for volume definition: surgical cavity plus the margin because it may be the site of resistant and rapidly proliferating clones (43).

Few studies have focused on the radiomic analysis of features in postoperative MRI. Dasgupta et al. generated probabilistic maps by developing a radiomic signature using imaging data from low-grade glioma (LGG) (tumor marker) and brain metastasis (BM) PTR (edema marker) and applied on 10 cases of GB PTR. They found that a radiomic signature can demarcate areas of microscopic tumors from edema in the PTR of GB, which correlates with areas of future recurrence. The authors finally suggested the potential application of radiomic features in driving radiotherapy target volumes, as standard practice includes a wider margin empirically (46).

Our study aimed to develop a predictive model based on radiomic features analysis extracted from real data to guide the target volume delineation in radiotherapy, focusing on the open question of the margins to be given to the surgical cavity, in order to re-evaluate and to hypothesize a CTV contouring guided and personalized according to radiomic features.

Considering our homogeneous population of 90 GB IDH wild-type, the analysis focused on a healthy tissue ring around the surgical cavity resulting in a radiomic model able to discriminate between patients with low-risk and high-risk of relapse at 6 months with an AUC of 78.5%. We decided to considerate the clinical outcome of PFS at 6 months that could describe the local control after radio-chemotherapy, excluding the overall survival that could depend on other clinical and treatment variables. This predictive model with high NPV of 0.84 could allow us to select a population of patients with low-risk of relapse at 6 months, in whom it may be possible to reduce the total CTV by decreasing the margins to 1.5 cm, planning a dose strategy modulation in the surrounding tissue and potential reducing the toxicity of healthy tissue and critical structures.

The radiomic features included in the developed radiomic model were textural features computed from the gray-level co-occurrence matrix (F_cm_merged.contrast, F_cm_merged.info.corr.2), which is based on the combinations of the gray-levels of neighboring pixels, and from the gray level run length matrix (F_rlm_merged.rlnu), which is based on the sequence of consecutive pixels with the same gray-level. Furthermore, the radiomic model presented a high NPV of 0.84 when compared to the null model, which was based on the prevalence of the majority class 0 (∼67%). This result was confirmed in the internal validation, which was performed to assess the generalizability of the model. The limitations of this study include the lack of independent validation of the proposed radiomic model, the absence of images for all patients due to unsuitable imaging data, small sample size and the lack of correlation with other potential clinical prognostic factors of PFS or with recurrence pattern.

However, this is the first hypothesis-generating study that applies a radiomic analysis based on the irradiated target volume as region of interest (ROI) for GB, focusing on healthy tissue ring around the surgical cavity on post-operative MRI. Future steps will include performing an external validation of the model and verifying the applicability of the model in the clinical practice through clinical trials.

## 4. Conclusion

This study provides a preliminary model for a decision support tool employing radiomic features for a customization of the radiation target volume in GB IDH wild-type in order to achieve a margin reduction strategy.

## Data availability statement

The raw data supporting the conclusions of this article will be made available by the authors, without undue reservation.

## Ethics statement

The studies involving human participants were reviewed and approved by Prof. Andrea Bacigalupo, Clinico Ematologo. Presidente Prof. Stefania Boccia, Biostatistico. Vicepresidente Dott. Paolo Angelo Bonini, Esperto di Bioetica Prof. Emilio Bria, Clinico–Oncologia Prof. Alessandro Caruso, Clinico–Ostetricia e Ginecologia Dott. Antonello Cocchieri, Rappresentante dell’area delle professioni sanitarie Dott. Alessio de luca, Farmacista SSR1 Dott. Francesco Filidoro, Farmacista esperto di Dispositivi Medici Avv. Danilo Gallitelli, Esperto in materia giuridica e assicurativa o Medico legale Prof. Fiorella Gurrieri, Esperto di Genetica Dott. Michele Lepore, Medico di Medicina Generale Prof. Giuseppina Loffredi, Rappresentante del Volontariato\Associazione Tutela Pazienti Prof. Camillo Marra, Clinico–Neurologia Dott.ssa Barbara Meini, Farmacista SSR1 Prof. Nadia Mores, Sostituto permanente del Direttore Sanitario Prof. Key Peris, Clinico–Dermatologo Prof. Giacomo Pozzoli, Farmacologo Prof. Riccardo Riccardi, Pediatra Prof. Dario Sacchini, Esperto di Bioetica. Membri esterni: Avv. Filippo E. Leone, Responsabile Grant Office Prof. Antonio Gioacchino Spagnolo, Esperto di Bioetica. The patients/participants provided their written informed consent to participate in this study.

## Author contributions

SC, IP, SG, GS, LB, MG, GD, GD’A, ML, and MC contributed to conception and design of the study. MC, FC, and FP organized the database. MC, FB, SC, RR, and RG performed Manual segmentation. SG and RR reviewed all cases’ Manual segmentation. HT, ND, CV, and DC performed the statistical analysis. MC and SC wrote the first draft of the manuscript. HT, MC, SL, and SC wrote sections of the manuscript. CA, AO, MB, CC, and VV participated in supervision. All authors contributed to manuscript revision, read, and approved the submitted version.
